# First quantitative dosages: Strong correlations between non-5-HT_2_Rs serotonin receptors on normal human heart valves

**DOI:** 10.3389/fcvm.2022.897657

**Published:** 2022-10-25

**Authors:** Olivier Schussler, Luc Maroteaux, Ramadan Jashari, Pierre Falcoz, Marco Alifano, Yves Lecarpentier, Jean-Marie Launay

**Affiliations:** ^1^Research Laboratory, Department of Cardiovascular Surgery, Faculty of Medicine, Geneva University Hospital, Geneva, Switzerland; ^2^Department of Thoracic Surgery, Hôpital Cochin, APHP, Paris, France; ^3^INSERM UMR-S 1270, Paris, France; ^4^Sorbonne Université, Paris, France; ^5^Institut du Fer à Moulin, Paris, France; ^6^European Homograft Bank, Brussels, Belgium; ^7^Department of Thoracic Surgery, Nouvel Hôpital Civil, Hôpitaux de Strasbourg, Strasbourg, France; ^8^AP-HP Centre, Université de Paris, Hôpital Cochin, Service Thoracique, Paris, France; ^9^Centre de Recherche Clinique, Grand Hôpital de l'Est Francilien, Meaux, France; ^10^INSERM-S 942, Centre for Biological Resources BB-0033-00064, University Paris, Hôpital Lariboisière, APHP, Paris, France

**Keywords:** serotonin receptor 5-HTRs, human heart valves, serotonin 5-HT, quantitative expression, valvular degeneration, heart valve disease

## Abstract

**Objectives:**

Although critical in animal and human development and pathology, a measurement of the quantitative expression of 5-HTR serotonin receptors on animal or human valvular tissues has never been performed.

**Methods:**

Quantification of the most frequent 5-HTRs reported as being present in human peripheral tissue was performed using radiolabeled agonists/antagonists. A membrane protein extract from normal human valves (aortic/mitral/tricuspid and some pulmonary) and associated diseased left myocardium, all unusable in clinics, were obtained from the Homograft bank.

**Results:**

We analyzed 5-HT_1A_R/5-HT_1B/D_R/5-HT_2A_R/5-HT_2B_R/5-HT _2C_R/5-HT_4_R/5-HT_7_R from 28 hearts. We confirmed the presence of tissue and measured the quantitative content for respective proteins in femtomol/mg of protein extracts: for 5-HT_2A_R (35.9+/−0.7), 5-HT_2B_R (28.8+/−1.3) but also a newly observed and robust expression for 5-HT_4_R (38+/−4.2). We identified one, 5-HT_1A_Rs (4.9+/−0.3), and the possible expression, but at a very low level, of previously reported 5-HT_1B/D_Rs (1.3+/−0.5) as well as the new 5-HT_7_Rs (3.5+/0.1) and 5-HT_2C_Rs (1.2+/−0.1). Interestingly, by using univariate analysis, we were able to observe many correlations between the different 5-HTR levels of expression especially between 5-HT_1A_R/5-HT_1B/D_R and also between 5-HT_4_R/5-HT_7_R, but none were observed between 5-HT_2A_R and 5-HT_2B_R. Using multivariate analyses for a specific 5-HTR level of expression, after adjustment for implantation sites and other 5-HTRs, we found that 5-HT_1A_R was correlated with 5-HT_1B/D_R;5-HT_4_R with 5-HT_7_R and 5-HT_1A_R;5-HT_2B_R with 5-HT_2A_R only. For 5-HT_2_C, no correlation was observed.

**Conclusion:**

5-HT_2A_R/5-HT_2B_R and 5-HT_4_R were all observed to have a high and equal level of expression on human valves, but that of 5-HT_1A_R was more limited. Since these non-5-HT_2_Rs are coupled with different G-proteins, with specific signaling, theoretically they may control the main 5-HT_2_R signaling (i.e., PLC/DAG-PKC-ERK/Ras/Src signaling) involved in valvular fibrosis and degeneration.

## Introduction

The role of increased serotonin (5-HT) signaling in heart valvular development and disease is of growing importance and interest (1–4). The implication of 5-HT was first described in patients with carcinoid diseases presenting valvular injuries secondary to increased circulation of 5-HT. A secondary effect observed was the valvular toxicity of serotoninergic receptor drug agonists (3). More recently, its role has been reported in myxoid valvular degeneration and mitral prolapse (MVP) (2, 3, 5). The common macroscopic and histological characteristics shared by drug-induced valvulopathy and acute rheumatic fever make it difficult to determine the involvement of 5-HT agonists in heart valve diseases (HVD) and to suggest the possibility of a similar serotoninergic mechanism. In carcinoid tumors, increased circulation of 5-HT leads to the formation of “carcinoid plaques” at the valve surface and corresponds to activation of valvular interstitial cells (VIC) and the deposition of glycosaminoglycans (GAG) within the extracellular matrix (ECM). These lesions are very similar to those observed in patients under serotonin receptor drug agonist treatment. Valvulopathies associated with various serotoninergic drugs have been shown to share a common feature in the form of activated 5-HT_2B_R receptors (5-HT_2B_R) (6–8). MVP is the most frequent heart valve disease affecting 2–3% of the population older than 65 years (9) and therefore millions of individuals in the world (9, 10). The pathophysiology of MVP involves “myxomatous degeneration”, defined as the accumulation of mucopolysaccharides and other ECM components and the activation of VICs that are responsible for the thickening and proliferative aspect of the valve tissue (3). It has been shown that 5-HT is locally secreted in the valvular cusps. One isoform of the enzyme involved in its synthesis, tryptophan hydroxylase 1 (TPH1), is enhanced by mechanical stimulation and in degenerative human myxomatous heart valves (11). The remodeling induced by these factors could be prevented by 5-HT_2B_R, 5-HT_2A_R, or TPH1 antagonists (11, 12).

Up to now, only the expression of the transcription factor for 5-HTRs has been investigated and not the true protein expression of the receptors (2, 3, 13). In humans with myxoid mitral valve regurgitation, observations have revealed an up-regulation by qPCR on mitral prolapse tissue for 5-HT_2A_R, 5-HT_2B_R, and TPH1, but a decrease in the serotonin transporter (SERT). However, at the same time, on histological sections, only 5-HT_2B_R staining is enhanced (2), but not the 5-HT_2A_R staining. This highlights the fact that qPCRs are not accurate in defining protein expression.

While some authors of the present article have considerable experience in the quantitative dosage of 5-HTRs in tissues (14–16), these dosages have never been performed simultaneously in any animal or on human tissues. With respect to cardiac valvular cusps, the quantitative dosage for any 5-HTR has never been performed on animal or human tissues.

In this study, we not only confirmed the presence of 5-HT_2A_R and 5-HT_2B_R on human heart valves that are critical in valvular pathology but also demonstrated that these two receptors are expressed at the same level. In addition, we reported, for the first time, the presence and abundance of 5-HT_4_R, which was observed to be at the same level as 5-HT_2A_R and 5-HT_2B_R. Beside these three main receptors, many 5-HTRs were expressed at low levels, such as 5-HT_1A_R and 5-HT_7_R, or very low levels, such as 5-HT_2C_R or the 5-HT1_B/D_R reported earlier (17). Most interestingly, we found very strong correlations between the quantitative expressions of the following pairs of non-5-HT_2_R serotonin receptors: 1) 5-HT_1A_R and 5-HT1_B/D_R_;_ 2) 5-HT_4_R and 5-HT_7_R. Unlike 5-HT_2A_R and 5-HT_2B_R that are coupled with a specific G-protein, Gq/G_11_ (18), and thus cannot control cAMP levels. 5-HT_1A_R and 5-HT1_B/D_R are coupled with G-protein, Gi/Go, and can thus potentially decrease cAMP levels (18). 5-HT_4_R and 5-HT_7_R are coupled with another G-protein, Gs (18), and can thus increase cAMP. By controlling the level of non-5-HT_2_R expression and possible subsequent signaling, it may therefore be possible to control the main 5-HT_2_R signaling activity involved in valvular pathology.

## Materials and methods

### Human specimens

All experimental procedures were carried out in accordance with the ethical standards of the responsible institutional and national committees on human experimentation, thereby respecting the Helsinki Declaration (1975). Normal human heart valves were obtained from the European homograft program in Belgium (19, 20). Patients or members of the patients' families gave their written consent. In this program, pulmonary valves and some aortic valves are generally used. In Brussels, for example, 50% of the pulmonary or aortic valves could not be used, mainly because of functional incompetence, morphological alteration, surgical cuts, or bacterial contamination (19, 21). Donors were younger than 55 years (20). A cardiac surgeon involved in the program collected only heart valve cusps and their associated left ventricles to be used as controls. Valvular cusps were put directly into three separate tubes for each valve and a piece of the associated left myocardium into three additional tubes and labeled. The project was approved by the institutional review boards of the University Hospital of Geneva, Switzerland [Approbation number CER: 12-150 (NAC 12-056)] and by a local committee at the European Homograft Bank in Brussels. After collection, the samples were preserved in N2 liquid vapor until transfer.

### Methods

Methods for membrane preparation have been previously reported (14) as well as the methods for membrane radiolabeling for specific 5-HTRs in other non-cardiac tissues (14, 15, 22).

### Reagents

#### [^3^H] radioligands and drugs

The specific ligands used in the study, purchased from Perkin-Elmer Life Sciences, were as follows: for 5-HT_1A_R: agonist 8-0H-DPAT; for 5-HT_1B/D_R: agonist GTI (Serotonin-5-O-carboxymethyl-Glycil-iodo-tyrosamine); for 5-HT_2A_R: antagonist MDL 100.97; for 5-HT_2B_R: antagonist LY26.6097; for 5-HT_2C_R: antagonist mesulergine; for 5-HT_4_R: antagonist GR113808; for 5-HT_7_R: antagonist Ly269970. The specificity of the different agonists or antagonists for each 5-HTR used in the study and the different results of membrane radioligand binding assays were discussed in an extensive review of the pharmacological consortium for 5-HTRs, in which one of the co-authors of the present study (Luc Maroteaux) was involved (23). In the radio-binding assay for each 5-HTR, the zero of fixation corresponded to the level of fixation obtained in the presence of the labeled 5-HTR agonist or antagonist and of very high amounts (i.e., 1 μM) of its unlabeled agonist or antagonist. In the radioligand binding assay, a positive signal above 5 fmol of radioligand binding per milligram of protein from the extract is considered to be true (23). Values of 0–5 fmol of this radio in femtomol/mg of protein extracts ligand binding per milligram may indicate that the binding is not in fact present.

#### Membrane preparation

To prepare crude membranes for binding assays, the membrane cusps were washed twice with cold PBS and then harvested with a rubber policeman in 1.5 ml of PBS containing 1 μg/ml pepstatin, 1 μg/ml antipain, 15 μg/ml benzamidine, and 0.1 mM phenylmethylsulfonyl fluoride, as described earlier (14). After centrifugation, the resulting pellet was frozen at −70°C before homogenization. The frozen pellet was thawed at 37°C, resuspended in 10 ml of cold EDTA, 1 mM EGTA, 0.1 mM phenylmethylsulfonyl fluoride, and a 10 mM imidazole buffer pH 7.30, then centrifuged for 10 min at 5,000 × g. The supernatant obtained from this centrifugation was collected, poured onto a 20% sucrose cushion, and then centrifuged for 90 min. at 100,000 × g. The pellet containing the membrane was then resuspended in 75 mM KCl 5 mM MgCl_2_ and a 1 mM EGTA 10 mM imidazole buffer pH 7.3 for use in binding assays. Protein contents were determined using the protein assay kit.

#### Membrane radioligand binding assay

The different radiolabeled agonists or antagonists were incubated with a membrane protein extract of fresh human valvular cusp membranes as reported in earlier publications for other tissues (14, 15, 22). The different radiolabeled agonists or antagonists for the 5-HTRs were as follows: for 5-HT_1A_R: partial agonist [^3^H] 80H-DPAT; for 5-HT_1B/D_R: [^125^I] GTI, for 5-HT_2A_R: antagonist [^3^H] MDL 100.97; for 5-HT_2B_R: antagonist [^3^H] LY26.6097; for 5-HT_2C_R: antagonist [^3^H] Mesulergine; for 5-HT_4_R: antagonist [^3^H] GR113808; for 5-HT_7_R: antagonist [^3^H] Ly269970.

Binding experiments were performed at room temperature and involved tissue shaking. Binding was initiated by the addition of 50 μl of 50 mM Tris Buffer, pH 7.40, containing 0.1–10 nM radiolabeled agonist or antagonist to 5-HTRs, or appropriate competing ligands to 50 μl of membrane (representing 20 μg of protein from heart valve extract). The preparations were incubated for 30 min at RT followed by the addition of 3 ml of ice-cold 50 mM Tris Buffer with a pH 7.40. Samples were filtered using polyethyleneimine-treated filters and counted. The specific binding was defined as the binding inhibited by 1 μM levels of unlabeled agonist/antagonist for each 5-HTR sub-type. All the experiments were performed in triplicate.

### Statistics

Mean comparisons between groups were performed using ANOVA. Correlations between quantitative variables were made using Spearman's Rank Correlation. Multivariate regression models were built to assess the independent relationships (each variable in relation to the others). Two types of models were used. In one type, we compared specific 5-HTR receptor levels with explicative variables being another specific single 5-HTR and the sites of implantation (i.e., aortic/mitral or tricuspid). In the second type, we analyzed specific 5-HTR receptor levels with the explicative variables being all the other 5-HTRs and the sites of implantation (i.e., aortic/mitral or tricuspid). A *P*-value < 0.05 was considered as statistically significant. Data processing and analysis were performed using the statistical software system SEM (SILEX Development, Mirefleurs, France).

## Results

### Quantitative expression of serotonin receptors on “normal” human heart valves and associated diseased left myocardium

Heart valve leaflets were harvested from donor heart recipients and comprised valves that were not suitable for transplantation. For all 28 hearts, we have at the same time tricuspid and mitral valves. For 14 of the 28 hearts, we also had the aortic valve and, for 3 of the 28 hearts, we had the four valves including the pulmonary valves. For all the 28 hearts, we also checked the expression of 5-HTRs in associated diseased left myocardium (i.e., mostly those having chronic cardiomyopathies, but with normal heart valves, or having an acute heart transplant dysfunction). All samples were collected from hearts obtained from patients younger than 55 years in the homograft program.

**Figure 1:**
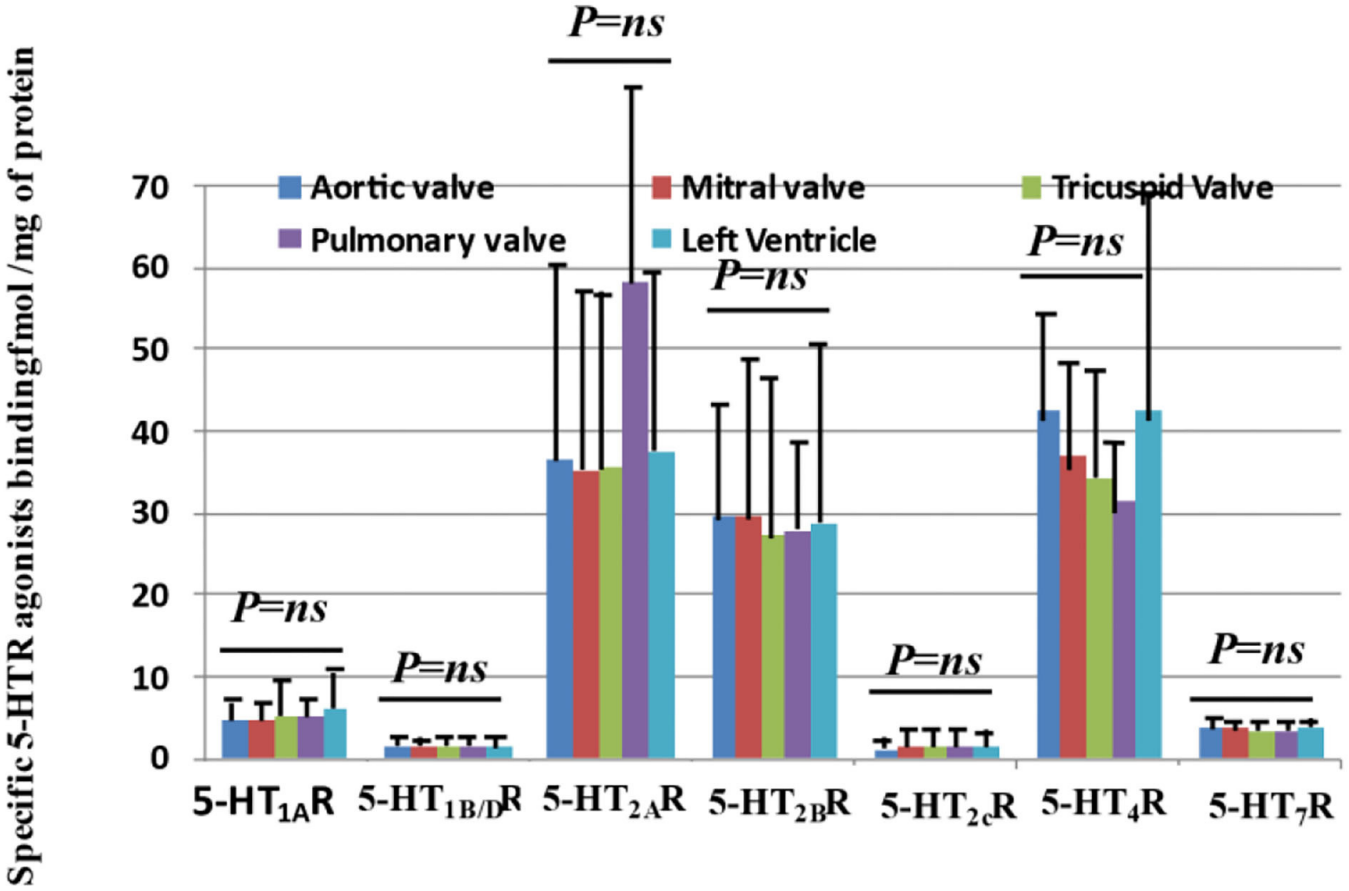
Quantitative expressions of 5-HTR receptors on human “normal” heart valves by radioligand binding affinity with the human left ventricle (i.e., diseased left cardiomyopathy) as a control. This figure shows the quantitative measurements of receptor expression for aortic, mitral, tricuspid, and pulmonary valves with the left ventricle as a control. Specific quantification of the protein expression for 5-HTRs (5-HT_1A_R, 5-HT1_B/D_R, 5-HT_2A_R, 5-HT_2B_R, 5-HT_2C_R, 5-HT_4_R, and 5-HT_7_R) on valvular tissue protein extract was performed with specific radioligands. 5-HT_1A_R and 5-HT_1B/D_R are known to be associated with a Gi/Go protein and thus decrease cAMP levels. On the other hand, 5-HT_4_R and 5-HT_7_R are associated with a Gs protein and thus increase cAMP levels. 5-HT_2_Rs are associated with another G-type protein (Gq/G_11_) and thus activate PLC, Src, and Ras. 5-HTR_7_R was the most recent receptor to be identified. Up until now, no quantification of 5-HTRs has been conducted on human heart valves. We had access to a total of 28 transplant hearts and had mitral and tricuspid sites for all of them. For 14 hearts, we had the three main sites (aortic/mitral/tricuspid), and for three hearts, we had four sites at the same time (including pulmonary site). All measurements of expression levels were performed in triplicate. As shown in this figure, all types of receptors we tested, known to be expressed in peripheral tissues, are expressed in valvular tissues, with the main receptors being quantitatively 5-HT_2A_R, 5HT_2B_R, and the unreported 5-HT_4_R. Interestingly, we did not observe any difference in terms of the level of expression between the different sides and sites investigated. Each bar diagram in the figure is based on 28 samples for tricuspid and mitral valves, with left ventricle as control, and 14 samples for the aortic position. *P*-values correspond to the results of ANOVA tests. No particular difference was observed between the right sides (i.e., tricuspid, pulmonary) and left sides (i.e., aortic, mitral) (ANOVA test). The pulmonary positions (i.e., only 3 samples available) tended to have the same level of expression as the other sites.

As shown in Figure 1, 5-HT_1A_R, 5-HT_1B/D_R, 5-HT_2A_R, 5-HT_2B_R, 5-HT_2C_R, 5-HT_4_R, and 5-HT_7_R are detected on human heart valve cusps. Among the different 5-HTRs tested, 5-HT_2A_R, 5-HT_2B_R, and 5-HT_4_R were quantitatively the most abundant (see Table 1 for mean values and SD). In this study, we confirmed the presence of 5-HT_2A_R and 5-HT_2B_R on human heart valves but also demonstrated that they were expressed in the same quantity. 5-HT_4_R has been reported in the myocardium but not on heart valves. The levels for 5-HT_2A_R/5-HT_2B_R/5-HT_4_R were around 30 femtomol/mg of proteins and thus very high (23).

**Table 1:** Quantitative expression of the different 5-HTRs (i.e., 5-HT_1_AR, 5-HT_1B/D_R, 5-HT_2A_R, 5-HT_2B_R, 5-HT_2C_R, 5-HT_4_R, 5-HT_7_R) on human valvular cusps (i.e., aortic *n* = 14, mitral *n* = 28, tricuspid *n* = 28 or pulmonary *n* = 3) or on human left myocardium as a control (*n* = 28) (see also Figure 1).

	**5-HT_1A_R**	**5-HT_1B/D_R**	**5-HT_2A_R**	**5-HT_2B_R**	**5-HT_2C_R**	**5-HT_4_R**	**5-HT_7_R**
Aortic, mean (SD)	4.8 (3.6)	1.3 (0.7)	36.7 (23.1)	29.5 (12.0)	1.1 (0.7)	42.7 (13.9)	3.7 (0.3)
Mitral, mean (SD)	4.7 (2.6)	1.4 (0.7)	35.5 (22.4)	29.7 (19.9)	1.3 (0.9)	37.1 (14.1)	3.5 (0.4)
Tricuspid, mean (SD)	5.3 (5.4)	1.4 (0.7)	35.5 (21.9)	27.2 (8.8)	1.2 (1.0)	34.3 (13.3)	3.5 (0.4)
Pulmonary, mean (SD)	5.2 (2.2)	1.5 (0.5)	58.1 (25.9)	27.9 (10.9)	1.5 (1.2)	31.3 (9.0)	3.4 (0.3)
Left myocardium, mean (SD)	6.1 (6.0)	1.4 (0.9)	37.4 (22.7)	28.9 (19.2)	1.3 (1.0)	42.3 (28.5)	3.6 (0.5)

For the first time, we reported the presence of one 5-HT_1_R receptor, the 5-HT_1A_R, at a low level (around 5 fmol/mg of proteins, or slightly above) which is nevertheless sufficient for specificity (23). We also detected a small amount of 5-HT_1B/D_R but at a very low level of around 1.3 femtomol/mg of proteins. Up to now, only 5-HT_1B/D_R (17) and not 5-HT_1A_R are present and functional on human heart valves (17).

Finally, we possibly detected a very low amount of 5-HT_7_R, around 3.5 femtomol/mg of proteins, and of 5-HT_2c_R around 1.5 femtomol/mg of proteins (23). Following univariate analyses, we did not observe any significant difference in the mean quantitative expression of specific 5-HTR receptors between the right and left side valves (Figure 1). When considering a particular patient and a specific 5-HTR, we only found a statistically positive correlation between the different locations for 5-HT_2B_Rs, but not for the other 5-HTRs. For 5-HT_2B_Rs, there were correlations between aortic and tricuspid valves (correlation 0.75 [0.17; 1.34]; *P* = 0.0042; *n* = 14; Spearman's Rank correlation) but not between aortic and mitral valves (correlation 0.077 [−0.38; 0.54]; *P* = 0.71; *n* = 28; Spearman's Rank correlation; Supplementary Figure S1). Surprisingly, the expressions of 5-HTRs in valvular cusps were very similar to those observed for the left myocardium ventricle that we used as a control (i.e., mostly obtained from patients with cardiomyopathies but with normal valves).

### Comparisons between quantitative expressions of different serotonin receptors on human heart valves, using univariate analyses

**Figure 2:**
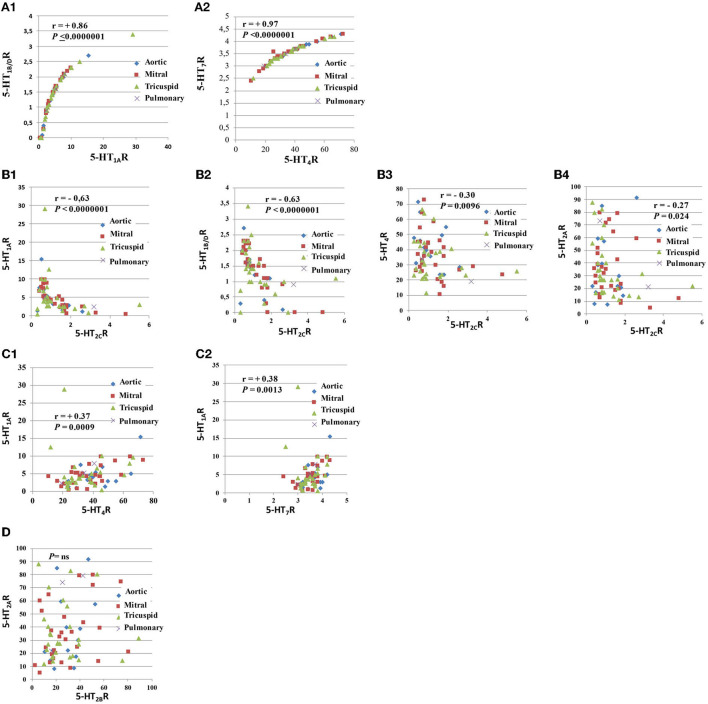
Comparison of the levels of expression of different pairs of 5-HTRs. This figure corresponds to this figure. As shown in this figure, there were strong correlations between several 5-HTRs **(A1–D)**. The strongest correlations were found between the 5-HT_1A_R and 5-HT_1B/D_R quantitative protein levels **(A1)** and between 5-HT_4_R and 5-HT_7_R levels **(A2)**. 5-HT_1A_R and 5-HT_1B/D_R are coupled with the same G protein (i.e., Gi/Go), while 5-HT_4_R and 5-HT_7_R are coupled with G protein Gs. We observed that 5-HT_2C_R levels were negatively correlated with all other 5-HTRs **(C)**. The negative correlation between 5-HT_2C_R levels and other 5-HT2Rs (i.e., 5-HT_2A_R or 5-HT_2B_R) was less marked **(B4)**. We also observed positive correlations between receptors coupled with different G proteins: 5-HT_4_R levels were negatively correlated with 5-H_1A_R levels **(C1)** and 5-HT_1A_R levels with 5-HT_7_R levels **(C2)**. There was no correlation between 5-HT_2A_R levels and 5-HT_2B_R levels after univariate analysis **(D)**. Respective correlations following univariate analyses are shown in different panels. All correlations between 5-HTRs following univariate analysis are presented in Table 2. Spearman's Rank Correlation analysis (*n* = 70 samples for each analysis).

As shown in Table 2, following a univariate analysis that included all the valves (except pulmonary; *n* = 70 = 14 + 28 + 28), we found numerous correlations between 5-HTR quantitative levels (i.e., Spearman's Rank correlation analysis). The highest correlations were found between 5-HT_1A_R and 5-HT1_B/D_R levels (*r* = +0.86; *P* < 0.0000001; *n* = 70; Figure 2A1) and between 5-HT_4_R and 5-HT_7_R levels (*r* = +0.97; *P* < 0.0000001; *n* = 70; Figure 2A2). 5-HT_2C_R was the only receptor to be negatively correlated with all the other 5-HTRs, including 5-HT_2A_R (*r* = −0.27; *P* = 0.024; *n* = 70) and 5-HT_2B_R (*r* = −0.23; *P* = 0.052; *n* = 70; Figures 2B1–B4). Using univariate analysis (Spearman's Rank Correlation; *n* = 70 samples for each analysis), we were unable to find any correlation between 5-HT_2A_R and 5-HT_2B_R levels (Figure 2D) or between 5-HT_2A_R or 5-HT_2B_R levels and other 5-HTRs, except with 5-HT_2C_R. However, we found a strong negative correlation between 5-HT_1A_R levels and 5-HT_4_R levels (*r* = +0.37; *P* = 0.0009; *n* = 70; Figure 2C1) and between 5-HT_1A_R levels and 5-HT_7_R levels (*r* = +0.38; *P* = 0.0013; n = 70; Figure 2C2; see also Table 2).

**Table 2:** Statistical correlations between the different 5-HTR receptors following univariate analysis.

**Factor 1; Factor 2**	**5-HT_1B/D_ R**	**5-HT_2A_R**	**5-HT_2B_R**	**5-HT_2C_R**	**5-HT_4_R**	**5-HT_7_R**
**Statistical comparisons between factor 1 and factor 2 following univariate analyses (*****n*** **=** **70)**						
5-HT_1A_R	***p*** **<** **0.0000001**	*p* = ns	*p* = ns	***p*** **<** **0.0000001**	***p*** **=** **0.0009**	***p*** **=** **0.0013**
	*r* = +0.863			*r* = −0.63	*r* = +0.37	*r* = +0.38
5-HT1_B/D_R		*p* = ns	*p* = ns	***p*** **<** **0.0000001**	***p*** **=** **0.00086**	***p*** **=** **0.013**
				*r* = −0.63	*r* = +0.39	*r* = +0.38
5-HT_2A_R			*p* = ns	***p*** **=** **0.024**	*p* = ns	*p* = ns
				*r* = −0.27		
5-HT_2B_R				*p* = 0.052	*p* = 0.083	*p* = ns
				*r* = −0.23	*r* = +0.21	
5-HT_2C_R					***p*** **=** **0.0096**	***p*** **=** **0.0038**
					*r* = −0.301	*r* = −0.34
5-HT_4_R						***p*** **<** **0.0000001**
						*r* = +0.97

r = correlation coefficient for the different univariate analyses SPERMAN Rank Correlation n = 70 for each specific analysis p = p-value.

Bold are statistical values.

### Multivariate regression analyses

Using multivariate regression analysis, we first compared the expression of a specific 5-HTR receptor and examined, as possible explicative variables, other specific 5-HTR levels and implantation sites (i.e., aortic, mitral, tricuspid; Table 3 and Supplementary Figure S2). We performed several different multivariate analyses and found the same type of significant correlations as revealed by univariate analysis. Each multivariate regression analysis was performed with 70 samples. Using multivariate regression analyses, after adjustment for implantation sites, 5-HT_1A_R levels were significantly correlated with 5-H_1B/D_R levels (coef. +0.54 [+0.49; +0.54]; *P* < 0.0000001). Similarly, 5-HT_4_R levels were found to be correlated with 5-HT_7_R levels (coef. +0.56 [+0.50; +0.62]; *P* < 0.0000001).

**Table 3:** Multivariate analysis after adjustment for another 5-HTRs for implantation sites (see also of Supplementary Figure S3).

**Factor 1; Factor 2**	**5-HT1_B/D_R**	**5-HT_2A_R**	**5-HT_2B_R**	**5-HT_2C_R**	**5-HT_4_R**	**5-HT_7R_**
Level of expression of factor 1 after adjustment for factor 2 and implantation site (aortic/mitral/tricuspid) following multivariate analyses*(aortic ***n*** **=** **14, mitral** ***n*** **=** **28, tricuspid** ***n*** **=** **28)**						
5-HT_1A_R	***p*** **<** **0.0000001**	*p* = ns	*p* = ns	***p*** **=** **0.0000019**	***p*** **=** **0.000055**	***p*** **=** **0.0045**
	Coef. = +0.54 [0.49; 0.54]			Coef. = −0.37 [−0.51; −0.23]	Coef. = +0.24 [0.12; 0.35]	Coef. = +0.10 [0.03; 0.17]
5-HT1_B/D_R		*p* = ns	*p* = ns	***p*** **=** **0.0000034**	***p*** **=** **0.00049**	***p*** **=** **0.01**
				Coef. = −0.67 [−0.93; −0.41]	Coef. = +0.37 [0.16; 0.57]	Coef. = +0.17 [0.04; 0.31]
5-HT_2A_R			*p* = 0.079	*p* = 0.11	*p* = ns	*p* = ns
			Coef. = +0.16 [−0.02; 0.34]	Coef. = −0.14 [−0.33; 0.03]		
5-HT_2B_R				*p* = ns	*p* = ns	*p* = ns
5-HT_2C_R					***p*** **=** **0.0068**	***P*** **=** **0.013**
					Coef. = −0.23 [−0.4; −0.06]	Coef. = −0.13 [−0.24; −0.03]
5-HT_4_R						***p*** **<** **0.0000001**
						Coef. = +0.56 [0.50; 0.62]

+Factor for adjustment in multivariate regression analyses after adjustment for another 5-HTRs and implantation sites.

Each table presents the results of multivariate regression analysis with the explicative variables being all the other 5-HTRs. Only models with significant interactions are reported. Each model includes 70 samples. The different coefficients determined by multivariate regression analysis are only reported for those with a p-value of ≤0.1. IC 95%: coefficient confidence level 95%.

The pulmonary valves (n = 3) are not included in the analyses due to their low number in the study.

Bold are statistical values.

**Figure 3:**
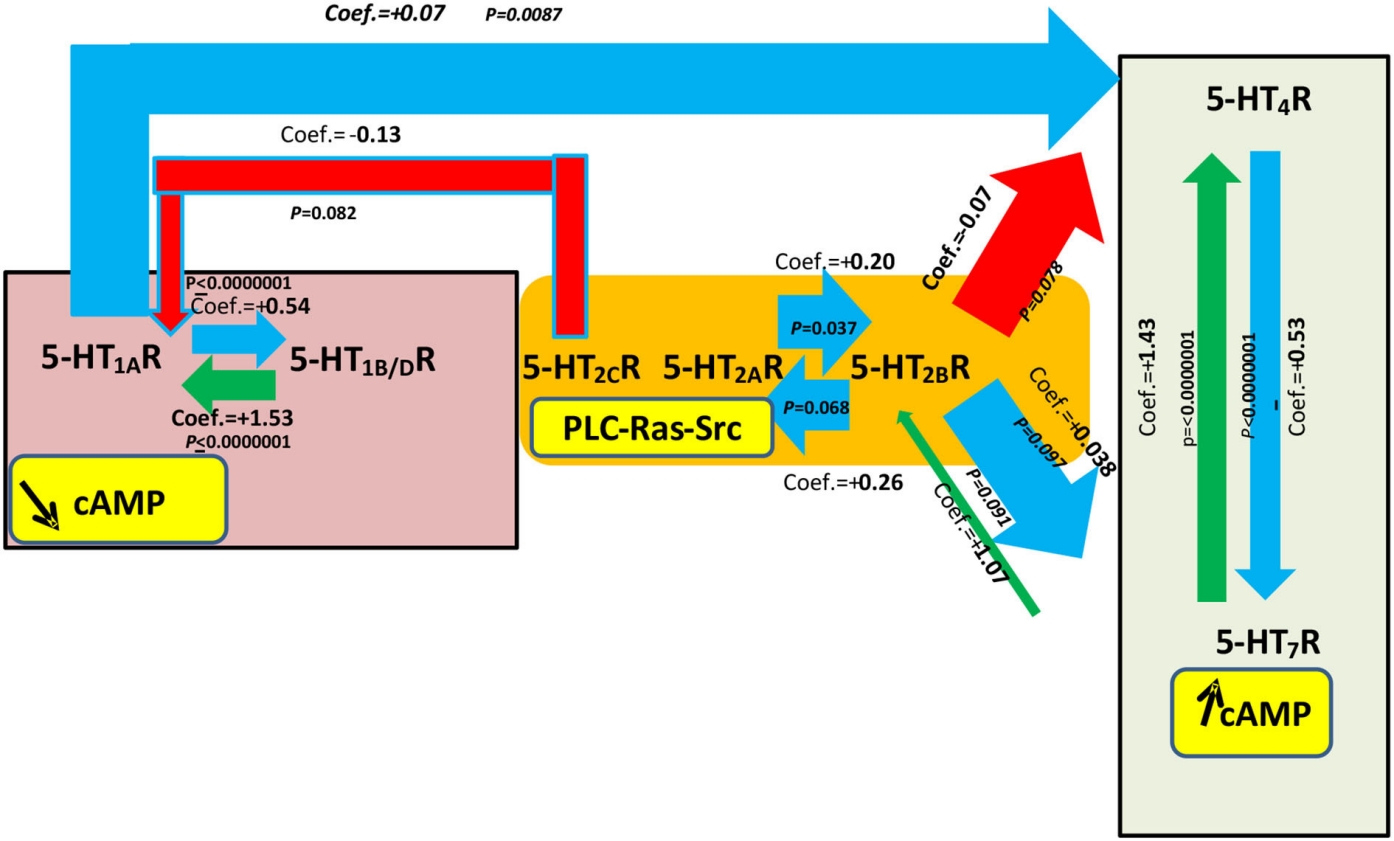
Most significant statistical associations based on multivariate regression analyses for a specific 5-HTR level of expression with explicative variables being all other 5-HTR receptors and the implantation sites. This figure is based on Table 4. Seventy samples were included in each statistical analysis. See also the corresponding Supplementary Figure S2 adapted from Table 3 corresponding to the most significant statistical associations after multivariate regression analysis between 5-HTR levels with the explicative variables being another 5-HTR and the site of implantation. The yellow boxes show the main classical signal transduction pathways that are possibly activated after stimulation from specific 5-HTRs: 5-HT_1A_R, 5-HT_1B/D_R, 5-HT_2C_R, 5-HT_2A_R, 5-HT_2B_R, 5-HT_4_R, and 5-HT_7_R especially with respect to the main signaling cAMP, but other signaling may also be present. The width of the arrows is proportional to the level of statistical correlation found following multivariate regression analysis. Only those comparisons between 5-HTRs with a *P-*value < 0.1 following multivariate regression analysis are shown in this figure. Blue arrows correspond to a correlation between 5-HTRs where the coefficient has an absolute value between 0 and 1 and is positive. Red arrows correspond to a correlation where the coefficient has an absolute value between 0 and 1 and is negative. In summary, as shown in this figure, statistically significant correlations were observed between levels for 5-HT_1A_R and 5-HT_1B/D_R and levels for 5-HT_4_R and 5-HT_7_R. For 5-HT4R levels, there was also a negative correlation with 5-HT1AR levels. Interestingly, we observed a negative correlation between 5-HT_2A_R levels and 5-HT_2B_R levels (coef. = +0.20 [−0.02; 0.54]; *P* = 0.037). The yellow boxes show the main signaling pathways for each 5-HTR if their specific G protein is involved. These signaling pathways are just hypothetical in heart valves and need confirmation.

In the second type of multivariate regression analyses (see Table 4 and Figure 3), we also compared the quantitative expression level for a specific 5-HTR, with the explicative variables being all the other 5-HTRs and the different implantation sites (i.e., aortic, mitral, tricuspid). Figure 3 shows only the significant interactions in our study, i.e., those with a *P*-value < 0.1 for the different multivariate analyses. The subsequent signaling (in yellow boxes) is only speculative. Each multivariate regression analysis included 70 samples. Correlation coefficients are reported only for *P-*values < 0.1. As in Table 4, following multivariate regression analysis, 5-HT_1A_R levels were independently correlated only with 5-HT_1B/D_R levels (coef. +1.53, [1.35; 1.7], *P* < 0.0000001). 5-HT_4_R levels were independently correlated with 5-HT_7_R levels (coef. +1.43 [+1.28; +1.58], *P* < 0.0000001) and 5-HT_1A_R levels (coef. +0.07 [+0.02; +0.12], *P* = 0.0087). 5-HT_2B_R levels were independently correlated with 5-HT_2A_Rs level only (coef. +0.20 [+0.01; +0.39]; *P* = 0.037).

**Table 4:** Factors controlling the expression of specific 5-HTR based on multivariate analysis after adjustment for all other 5-HTRs and implantation sites (see also Figure 4).

**Principal variable + Factors for adjustment in multivariate analysis**	**Coef**.	**IC 95%**	***p*-value**
	**5-HT**_**1A**_ **R**	**IC 95%**	***p*** **-value**
5-HT_1B/D_R	1.53	[1.35; 1.7]	*p* < 0.0000001
5-HT_2C_R	−0.13	[−0.27; 0.01]	*p* = 0.082
	**5-HT**_**1B**_	**IC 95%**	***p*** **-value**
5-HT_1A_R	0.54	[0.49; 0.59]	*p* < 0.0000001
	**5-HT**_**2A**_	**IC 95%**	***p*** **-value**
5-HT_2B_R	0.26	[−0.02; 0.54]	*p* = 0.068
	**5-HT**_**2B**_	**IC 95%**	***p*** **-value**
5-HT_2A_R	0.20	[0.01; 0.39]	*p* = 0.037
5-HT_7_R	1.07	[−0.17; 2.33]	*p* = 0.091
5-HT_4_R	−0.6	[−1.38; 0.16]	*p* = 0.12
	**5-HT**_**4**_	**IC 95%**	***p*** **-value**
5-HT_7_R	1.43	[1.28; 1.58]	*p* < 0.0000001
5-HT_1A_R	0.07	[0.02; 0.12]	*p* = 0.0087
5-HT_2B_R	−0.06	[−0.12; 0.01]	*p* = 0.078
	**5-HT**_**7**_	**IC 95%**	***p*** **-value**
5-HT_1A_R	−0.42	[−0.56; −0.27]	*p* < 0.0000001

+Factor for adjustment in multivariate regression analyses for all other 5-HTRs and implantation sites.

Each table presents the results of multivariate regression analysis with the explicative variables being all the other 5-HTRs and the implantation sites. Only models with significant interactions are reported. Each model includes 70 samples. The different coefficients determined by multivariate regression analysis are only reported for those with a p-value of ≤0.1. IC 95%: coefficient confidence level 95%.

Only factors with a p-value of below 0.1 in multivariate analyses are reported.

One limitation of the study is that we just did check for correlations and but we did not check for causality. If the correlations are a result of direct causality and some of the 5-HTRs can be used to control for others, it might then be possible to explain the correlations through physical interaction between the different 5-HTRs in valvular tissue. Even if the different receptors are not present in the same cells, they can still interfere with other 5-HTR signaling. Regulation of the membrane expression of 5-HTRs may be a way of controlling their specific signaling. Putative mechanisms linking the level of expression of different 5-HTRs on heart leaflets and possible secondary signaling, especially with regard to their respective known associated G proteins, are presented *in* Supplementary Figure S3.

## Discussion

In this study, we confirmed the expressions of 5-HT_2A_R and 5-HT_2B_R on human heart valve leaflets and, interestingly, also found that they were quantitatively very similar. We also reported, for the first time, the presence of 5-HT_4_R and at a very high level and in the same quantity as for 5-HT_2A_R and 5-HT_2B_R. 5-HT_2A_R and 5-HT_2B_R are known to be coupled with a specific G protein, Gq/G_11_, and thus cannot directly control the cAMP levels, while 5-HT_4_R are coupled with another *G* protein, *Gs*, (*s* = that stimulates adenylate cyclase activation), resulting in an increase in the intracellular cAMP concentration. Furthermore, we found a lower amount of 5-HT_1A_R and very low amounts of 5-HT_7_R, 5-H_1B/D_R and 5-H_2C_R. 5-H_1_Rs are coupled with another *G* protein: *Gi*/*G*0 (“*i*” for inhibiting the adenylate cyclase adenylyl cyclase) and thus potentially decrease the cAMP levels, while 5-HT_7_Rs, like 5-HT_4_Rs, are coupled with Gs, and thus increase cAMP levels (18). To date, apart from 5-HT_2A_R and 5-HT_2B_R, only 5-H_1B/D_R (which has a very low level of expression in our study) has been shown to be present and functional on heart valves (17).

### First quantitative dosage of 5-HT_2A_R and 5HT_2B_R on “normal” human leaflets

**Figure 4:**
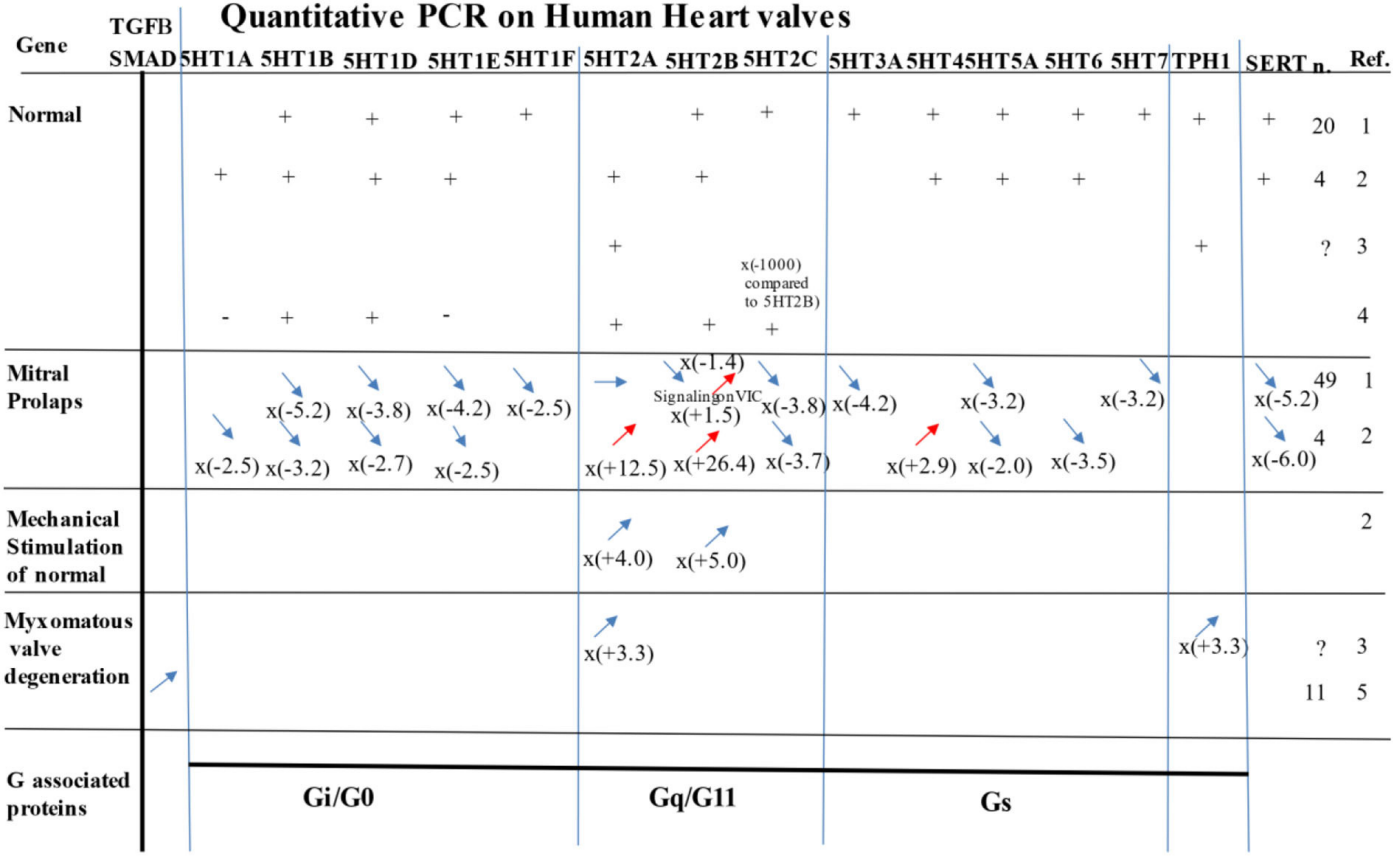
Most significant study results evaluating expression of 5-HTRS, TPH1, or SCC6 (i.e., the gene for SERT) by PCR on human samples in normal, prolapse, myxomatous degenerative valves, or under mechanical stimulation. *n* = number of samples analyzed. For PCR quantification for mitral prolapse and myxomatous valve degeneration, the values are compared to the normal. Ref. references for this figure/ 1) Levy et al. (5); 2) Driesbaugh et al. (2); 3) Hulin et al. (25); 4) Roy et al. (27); 5) Sainger et al. (28). See also for review: Ayme-Dietrich et al. (3) and Barnes et al. (11, 23).

5-HT_2A_R, 5-HT_2B_R (2, 6), and possibly 5-HT _1B/D_R (17, 24) have been shown to play a role in human valvular pathology. In this study, we confirmed the presence of 5-HT_2A_R and 5-HT_2B_R in human valvular leaflets but also observed that both were present at a very high level and in the same quantities, suggesting that both receptors may also play an important role in humans. Evidence of the involvement of serotonin in valvular heart diseases, such as in degenerative myxoid heart valves, is increasing (3). As with mice, recent studies using microarray technology highlight the contribution of 5-HT_2B_Rs in pathological conditions, such as human myxomatous mitral disease (25) (for summary, see Figure 4). Recently, using qPCR, 5-HT_2B_Rs and 5-HT_2A_Rs have been reported as being over-expressed in human mitral valve prolapse, but only the 5-HT_2B_R protein was found to be increased in histological sections (2). Over-expression of mRNA for 5-HT_2A_R (x12) and 5-HT_2B_R (x28) has been observed on human heart valves in prolapses (2). However, another study using qPCR has not confirmed an increase in 5-HT_2B_R in human valvular prolapse (5). 5-HT_2B_R may also play a crucial role in the propensity of valvular tissue to develop calcification. Antagonists for 5-HT_2B_R have been shown to prevent aortic valve calcification by inhibiting VIC through physically arresting Src-tyrosin-kinase (26). In humans, as with mice, TPH1, the enzyme involved in its peripheral synthesis of serotonin (5-HT), is locally secreted in valvular leaflets and may play a crucial role in pathology. *In vitro*, its transcription is increased by mechanical stimulation only (12). *In vitro*, specific antagonists for 5-HT_2A_R, 5-HT_2B_R, and TPH1 also block the valvular remodeling induced by mechanical stimulation (12). *In vivo*, transcriptions for TPH1, 5-HT_2A_R, and 5-HT_2B_R are increased during valvular pathology (12).

### First quantitative dosage of non-5-HT_2_R (serotonin receptors) on “normal” human heart valves

In this study, we found a very high level of 5-HT_4_R as well as some 5-HT_1_R (i.e., 5-HR_1A_R) that had never been reported before. Recently, qPCR has been used in human adult leaflets, taking into account the limits of the technique because of possible alternative splicing (3, 13), to observe mRNA expression for 5-HT_1A_R, 5-HT_1B/D_R, 5-HT_2A_R, 5-HT_2B_R, 5-HT_2C_R, 5-HT_4_R, 5-HT_5_R, 5-HT_6_R, and SERT (2) (see Figure 4).

In the present study, we also reported a robust high quantitative protein expression of 5-HT_4_R on human leaflets. Until now, 5-HT_4_R has only been reported in the human myocardium and in association with atrial arrhythmias (29–32).

We reported the presence of several 5-HT_1_Rs (i.e., 5-HT_1B/D_R and 5-HT_1A_R) with a higher amount of 5-HT_1A_R than 5-HT_1B/D_R. Up to now, only the 5-HT_1B/D_Rs (3, 13) have been reported in human valve leaflets, but not the 5-HT_1A_Rs. 5-HT_1B/D_Rs have also been shown to play a role in valvular pathology (17, 24). In our study, we reported a very low level of expression for other 5-HT_2_Rs (i.e., 5-HT_2*C*_R). Earlier studies using the qPCR technique found a very low level of transcription for 5-HT_2_C (compared with 5-HT_2A_R or 5-HT_2B_R) in valvular tissue (−300x) (27).

Carcinoid tumors are known to be more often associated with valvular heart diseases on the right side. Most (90%) of the 5-HT synthesized in the body comes from the periphery, where it is mainly produced by gut enterochromaffin cells from the essential amino acid, tryptophan, and the limiting enzyme, tryptophan hydroxylase-1 (TPH1), and then taken by SERT and stored in platelet dense granula (33). After release, the 5-HT will thus predominantly react with the right-side valves before being degraded in the pulmonary circulation (33). This is also in agreement with observations in humans with the unusual involvement of valvular diseases of the left side, when an anormal patent foramen is present, enabling blood from the right side to directly interact with the left heart valves. In animals, intravenous administration of 5-HT has been shown to be associated with right and left side diseases, while in humans some serotonin drug agonists have been shown to be associated with diseases on both sides. In our experiments, we did not find any differential expression for various 5-HTRs between the left and right sides. This study confirmed that it was rather the spatial distribution of the agonist 5-HT and not the spatial distribution of 5-HTRs between leaflets that explained the specific leaflet involvement. Our data are also in agreement with valvular development, in that during development, 5-HTRs are mainly expressed by neural crest cells, while SERT is expressed by secondary-heart-field cardiomyocytes. Both these cell types will be redistributed equally on the right and left side leaflets and thus 5-HTRs should be equally distributed between the different leaflets (34).

We reported strong correlations between the quantitative expressions of non-5-HT_2_R serotonin receptors on normal human heart valves.

We also observed very strong correlations between the quantitative expressions of 5-HT_1A_R and 5-HT_1B/D_R, and between 5-HT_4_R and 5-HT_7_R. We found that many non-5-HT_2_Rs had levels of membrane expression that were closely correlated with the level of other non-5-HT_2_Rs. Such a complex regulatory relationship has not been shown so far in any tissue. The highest correlations were observed between the quantitative expressions of proteins of 5-HT_1A_R and 5-HT_1B/D_R and between the quantitative expressions of proteins of 5-HT_4_R and 5-HT_7_R. While we had expected to find some correlations between 5-HT_2A_R and 5-HT_2B_R, we found none. Thus, it seems most probable that 5-HT_2_Rs, which are crucial for terminal valvular functionality and pathology, do not regulate each other.

The strongest correlations between 5-HTRs were found between 5-HT_1A_R and 5-HT_1B/D_R, both of which are associated with the same *G* protein (*Gi*/*G*0) and can thus decrease the cAMP levels, and between 5-HT_4_R and 5-HT_7_R, which are associated with another *G* protein, *Gs*, and can thus increase the cAMP levels (18).

### Explaining the different correlations between 5-HTR levels

Thanks to the quantitative assays we performed, revealing significant differences between the levels of 5-HTR expression on human heart valve tissues, it would seem that the regulatory mechanisms of 5-HTR expression are probably not unique. As we discussed, several cell types are present in valvular leaflet preparation (i.e., endothelial cells, mesenchymal cells/fibroblasts/myofibroblasts, valvular interstitial cells). The control of the regulation of 5-HTRs between each other may not necessarily require physical contact between the different 5-HTRs. If the different receptors are on the same cells, they might form homodimers or interfere with other 5-HTR signaling or levels of expression. Heterodimerization can theoretically explain the tight correlations between the levels of 5-HTRs with the same levels of expression (i.e., with a high or a low level) such as for 5-HT_1A_R and 5-HT_1B/D_R that have the same low levels in our study. At the same time, heterodimerization cannot explain the strong correlations observed between 5-HT_4_R and 5-HT_7_R, which had levels of quantitative protein expression that were very different. Thus other mechanisms are most likely involved.

Possible heterodimerization between non-5-HT_2_Rs for the regulation of their respective quantitative levels of expression has been reported in the literature for some tissues.

Heterodimerization requires that 5-HTRs are on the same cell and that there is some physical contact between 5-HTR monomers.

5-HTRs are present at the cell surface in a dynamic equilibrium with constant formation and dissociation of new receptor complexes (35). 5-HT_2A_R/5-HT_2C_R can function as stable homodimers, but 5-HT_2B_R homodimers have not yet been found (36). Dimers have also been documented for 5-HT_1A_R/5-HT_1B/D_R/5-HT_4_R/5-HT_7_R (36).

In cells in non-cardiac tissue, heterodimerization has been shown between 5-HT_1B_R and 5-HT_1D_R, 5-HT_1A_R and 5-HT_7_R, 5-HT_1A_R and 5-HT_2A_R, 5-HT_2A_R and 5-HT_2B_R, and, for 5-HT_2C_R, only with 5-HT_2A_R or 5-HT_2B_R (37).

A physical dimerization has been shown (38) between 5-HT_1B_R and 5-HT_1D_R, but not between 5-H_1A_R and 5-HT _1B/D_R. Heterodimerization between Gi/0- and Gs-coupled receptors has also been reported, while heterodimerization between 5-HT_1A_R and 5-HT_7_R, has been demonstrated with inhibition of 5-HT_1A_R signaling (37). Heterodimerizations between *Gi*/0 and *Gq* have also been reported between 5-HT_1A_R and 5-HT_2A_R (37). 5-HT_2B_R heterodimerization with 5-HT_1B/D_R has been shown to increase 5-HT_1B/D_R internalization after 5-HT_2B_R stimulation with a specific agonist (37).

In cardiac fibroblasts, heterodimerization between 5-HT_2B_R and non-5-HTRs (i.e., angiotensin II) has been reported. In this experiment, in which one of the authors of the present study was involved, stimulation by angiotensin II promoted hypertrophy (39).

Besides homodimerization, in non-cardiac tissues, other complex signaling regulatory networks have been shown to allow, for a specific 5-HTR, the control of other 5-HTR expression levels.

On the same cell, but without the physical contact that is required for “heterodimerization”, the 5-HTRs can still theoretically control their respective levels of expression or signaling.

A general feature of GPGRs (i.e., “G-protein-coupled receptor families”) (35) such as 5-HTRs is the existence of complex intracellular regulatory mechanisms that modulate the receptor responsiveness. Receptor desensitization and down-regulation are well documented and are important for homeostatic mechanisms. Homologous desensitization occurs when a receptor decreases its response to an agonist at high concentrations. Heterologous desensitization involves desensitization after stimulation of another receptor. The serotonin agonist itself has been shown to provoke internalization of 5-HT_2A_R, 5-HT_2B_R, and 5-HT_2C_R. Stimulation of 5-HT_1B_R or 5-HT_2B_R without physical interaction has been shown to affect the internalization dynamics in a heterologous manner, especially for 5-HT_2B_R (15). It has been shown that the co-expression of 5-HT_2B_R with 5-HT_1B_R induces a marked acceleration of 5-HT_1B_R internalization, without direct physical interaction (15). Thus, 5-HT_2B_R can control and diminish 5-HT_1B/D_R membrane expression levels by inducing their internalization.

Serotonin signaling is known to be one of the few key pathways involved in human myxoid valve degeneration.

Heart valves are dynamic multilayered structures that are actively remodeled by activation of the main cellular type, the valve interstitial cells (VICs). In adults, VICs are quiescent but become activated by mechanical stimuli.

*In vitro*, on human mitral valvular interstitial cells, it has been shown that mechanical stress induces an early and transient TGF-β2, αSMA, and CTGF (i.e., profibrotic growth factor). The signaling pathways are RhoC/ROCK/MRTF-A and ERK1/2 (40). The 5-HT_2B_R antagonists do not inhibit the canonical TGF-β/smad3 phosphorylation but prevent the non-canonical p38 MAPK phosphorylation by physically arresting Src (26). With the progression of valvular disease, there is a progressive shift from initial canonical TGF-β pathways (Smad 2/3/4) to TGF-β (Smad 5/6/7), BMP, and the canonical Wnt signaling (41). The myxoid heart valve has been related to serotonin (2, 42), angiotensin II (43), and activation of TGF-β pathways (44). In humans, several studies have shown a possible link between serotonin 5-HT dysregulation and the development of MVR. It has been shown that 5-HT is locally secreted in valvular cusps and the enzyme involved in its synthesis, the TPH1 is enhanced in the degenerative human myxomatous heart valve (11). In humans, recent observations have revealed an up-regulation of RNAs for 5-HT_2A_R and 5-HT_2B_R (2). *In vitro*, tissue leaflet remodeling can be prevented by an antagonist for 5-HT_2A_R or 5-HT_2B_R only, or an inhibitor of TPH1 (12). Today, it is increasingly recognized that mechanical stress is a major etiological factor underlying soft connective tissue remodeling, including pathological MVP. Various studies in animal and human hearts with aortic and mitral valve leaflets have shown that mechanical stimulation is associated with VICs activation toward SMC phenotypes, increased synthesis of PGs, GAGs, and collagen as well as an increased expression of proteolytic enzymes.

In humans with myxomatous degenerative mitral heart valves (*n* = 11), on the regurgitating leaflet, an up-regulation of RNA expression was observed on mitral prolapse tissue for 5-HT_2A_R (12x) and 5-HT_2B_R (28x), and a decrease in 5-HT_1A_R (−2.5x), 5-HT_1B/D_R (−2.7x), and 5-HT_2C_R (−3.7x). There was also an increase in 5-HT_4_R (2.9x) and TGF-β2 (3x) and a decrease in SERT (−6x; see Figure 4). Another recent study, using also qPCR in patients (*n* = 44) with MV prolapse, has shown no up-regulation of 5-HT_2A_R, while 5-HT_2B_R was even down-regulated: (−1.4x), 5-HT_2C_R (−3.9x), 5-HT_1B_R (−5.5x), 5-HT_1D_R (−3.1), 5-HT_7_R (−3.2x), and SERT (−5.2x). In this study, TPH1 has been shown to be down-regulated (−2.3x) but 5-HT_1A_R and 5-HT_4_R were not significantly changed (5) (see Figure 4). TGF-β2 was again increased (2.3x), especially BMP4 (+2.0x), but not BMP3 (−2.7x) or BMP5 (−4.9x). The blockers of SERT *in vitro* increased the 5-HT_2B_R on VIC (5).

Regulation of the level of specific non-5-HT_2_Rs and subsequent signaling, especially regarding cAMP signaling, might theoretically be a way of interfering with valvular pathological progression.

The 5-HT_2A_R and 5-HT_2B_R signaling is essential in human valvulopathy and involves activation of Gq/G11 proteins and subsequent PLC/DAG-PKC-ERK/Ras/Src signaling. 5-HT_2A_R and 5-HT_2B_R, in general tissue in animals and humans, are associated with increased fibrosis. In animal and human heart valves, 5-HT_2A_R and 5-HT_2B_R are also associated with valvular fibrosis (3). Among the different 5-HTRs, only HT_2A_R and 5-HT_2B_R have been shown, in both animals and humans, to be associated with valvular pathology (3). None of the other 5-HTRs have been shown to have such a capacity (3). In mice and humans, 5-HT_2A_R and 5-HT_2B_R, and subsequent PLC/DAG-PKC-ERK/Ras/Src signaling, have been reported to be associated with valvular fibrosis and, among different 5-HTRs, are the key 5-HTRs involved in the degeneration process (2, 5, 11, 12, 23, 42). In animal and human myxomatous degenerative valves, the local secretion of serotonin is also increased with increased TPH1 (11). We hypothesize that this signaling might be controlled by the regulation of non-5-HT_2_Rs in particular (i.e., 5-HT_1A_R, 5-HT_1B/D_R, 5-HT_4_R, and 5-HT_7_R), which can regulate the cAMP levels. The role of cAMP is to inhibit ERK1/2 and Smad 2/3/4 and to stimulate Notch and PKA, a role that has been reviewed recently (45). By acting on cAMP levels, the non-5-HT_2_Rs may promote or reduce fibrosis.

## Limitations of the study

One limitation of the study is that the levels of expression for 5-HTRs, other than 5-HT_2A_R, 5-HT_2B_R and 5-HT_4_R, were very low, below the standard detection level for pharmacology assays that is around 5 fmol of ligand binding per mg of protein extracts (23). With respect to 5-HT_1A_Rs, their levels of expression were 5.3 (±5.4) fmol/mg of protein in tricuspid position and 4.7 (+/2.6) fmol/mg of protein in mitral position (23).

In our study, 5-HT_1B/D_Rs presented one of the lowest levels of expression, with a level of around 1.4 (±0.7) fmol/mg of protein for the different valvular positions and thus far below the 5 fmol/mg level of proteins_._ However, its true presence and functionality have been proven by other groups in mice (24) and human fibroblasts derived from human heart valves (17).

In recent studies comparing normal human valvular leaflets and regurgitating leaflets, RNA expression was observed on normal valve and mitral prolapse tissues for most 5-HTRs that we identified in this study: 5-HT_1B/D_R, 5-HT_1A_R, 5-HT_2A_R, 5-HT_2B_R, 5-HT_2C_R, and R 5-HT_4_R (2, 4).

The best correlations we found were between 5-HT_1A_R and 5-HT_1B/D_R and between 5-HT_4_R and 5-HT_7_R. Since we are below the theoretical level of 5 fmol/mg of proteins for 5-HT_1A_R, 5-HT_1B/D_R, and 5-HT_7_R, we cannot exclude that for these receptors the interactions with the specific agonist may not be real. In a recent study on human mitral prolapse compared to human normal valve, the variations for 5-HT_1A_R and 5-HT_1B/D_R are almost the same [i.e., (−2.5x) and (−2.7x)], suggesting that a possible close regulatory relationship between these receptors in human pathology may exist (2, 4).

Another important limitation of the study is that we performed the analysis on an extract taken from the valvular tissues and not on a specific cellular population subtype. Heterodimerization between serotonin receptors requires that 5-HTR receptors be on the same cell. We cannot prove this. At the same time, in human valvular tissues, the expression of 5-HTR has so far been shown to be limited to a few cell types in valves (i.e., endothelial cells, fibroblasts/myofibroblasts, and interstitial valvular cells) (2, 17). The overall idea of having possible crosstalk between different cell types to regulate the level of expression of serotonin 5-HT receptors is still valid at the level of the overall preparation.

In this study, we did not specifically investigate signaling proteins in the preparation for cAMP or other signaling and all the mechanisms evoked were merely putative. Considering the 5-HTRs, it is known that their main signaling mechanism is due to their associated G proteins (18). At the same time, signal transduction following 5-HTR stimulation by agonists is not limited to the signal associated with their G protein, and each 5-HTR has a specific signal (18). 5-HT_1_Rs inhibit the adenylate cyclase (AC) and thus decrease cAMP. Both 5-HT_1A_R and 5-HT_1B/D_R stimulate ERK. In addition, 5-HT_1A_R frequently activates the K+ channel and inhibits the Ca^2+^ conductance. There are other pathways such as PLC and NOS (18). 5-HT_7_R activates AC and PKA as well as ERK (18). With respect to 5-HT_2A_R and 5HT_2B_R, they have the same main signaling: PLC, ERK, and PLA2. However, 5-HT_2A_R also activates PKC, and is responsible for the activation or inhibition of AC, and the activation of Jak2, Stat3, and Ca^2+^ channels. 5-HT_2B_ can also activate the cell cycle and iNOS. Like other 5-HT_2_Rs, 5-HT_2B_ can also activate ERK1/2. Finally, 5-HT_4_R can activate AC and various channels.

Another limitation of our study is that we only identified correlations between 5-HTRs and did not check for possible causality. This should be evaluated in future studies.

## Conclusion and future directions

In conclusion, our study reveals that many 5-HTR proteins are present in the extract of “normal” human valvular leaflets, especially 5-HT_2A_R, 5-HT_2B_R and the newly reported 5-HT_4_R, all three of which were observed at similar levels. There is also a smaller amount of 5-HT_1A_R and a possible expression of 5-HT_1B/D_ R and 5-HT_2C_R, but at even lower levels. All these 5-HTRs are known to be linked to specific *G* proteins: *G*_*i*_/*G*_0_, *Gq*/*G*_11_, or *Gs*. Interestingly, very strong correlations were found between non-5-HT_2B_R levels, especially between 5-HT_1A_R and 5-HT_1B/D_R and between 5-HT_4_R and 5-HT_7_R. 5-HT_1A_R and 5-HT_1B/D_R are associated with the same G-protein (*Gs*) and can thus increase the cAMP in valvular tissue, while 5-HT_1A_R and 5-HT_1B/D_R are coupled to another *G* protein (*G*_*i*_/*G*_0_) and can thus theoretically decrease cAMP. All these signaling mechanisms, and particularly their associated proteins, need to be assessed, If other groups can show causality, rather than just the presence of correlations between expressions of 5-HTRs, as we have done in this study, the regulation of the level of expression of non-5-HT_2_Rs, and subsequent signaling, might be a way to control the signaling activity of 5-H_2B_R and 5-HT_2A_R, which are the main serotonin receptors involved in animal and human pathology (i.e., PLC, ERK1/2, IP3, Ca^2+^, Src, Ras, and TGF-β).

## Data availability statement

The original contributions presented in the study are included in the article/Supplementary material, further inquiries can be directed to the corresponding author.

## Ethics statement

The studies involving human participants were reviewed and approved by Human Specimens. All experimental procedures were done in accordance with the ethical standards of the responsible institutional and national committee on human experimentation, adhering to the Helsinki Declaration (1975). Patient or patient's family gave their written consent to the program of homograft. The project was approved by the Institutional Review Boards of the University Hospital of Geneva, Switzerland [Approbation number CER: 12-150 (NAC 12-056)] and by a local committee at the European Homograft Bank in Brussels, Belgium. The patients/participants provided their written informed consent to participate in this study.

## Author contributions

OS, LM, and YL: design. RJ: samples collection. J-ML: quantitative dosages. OS and MA: statistics. All authors participate in the drafting of manuscript, read, and approved the submission of the manuscript.

## Conflict of interest

The authors declare that the research was conducted in the absence of any commercial or financial relationships that could be construed as a potential conflict of interest.

## Publisher's note

All claims expressed in this article are solely those of the authors and do not necessarily represent those of their affiliated organizations, or those of the publisher, the editors and the reviewers. Any product that may be evaluated in this article, or claim that may be made by its manufacturer, is not guaranteed or endorsed by the publisher.
