# Supplementary material for: First quantitative dosages: Strong correlations between non-5-HT2Rs serotonin receptors on normal human heart valves
Source: Front Cardiovasc Med. 2022 Oct 25;9:897657. doi: 10.3389/fcvm.2022.897657 (PMC9641165; doi:10.3389/fcvm.2022.897657)
Supplement: Supplementary file 1 [file Data_Sheet_1.pdf]

SUPPLEMENTARY MATERIAL

First quantitative dosages: strong correlations between non-5-HT_2Rs serotonin receptors on normal human heart valves

Olivier Schussler[1,2,*], Luc Maroteaux[3], Ramadan Jashari[4], Pierre Falcoz[5],

Marco Alifano[6], Yves Lecarpentier[7], Jean-Marie Launay[8]

[1] Department of Cardiovascular Surgery, Research Laboratory, Geneva University Hospital, Faculty of Medicine, Geneva, Switzerland

[2] Department of Thoracic Surgery, Hôpital Cochin, APHP, Paris, France

[3] INSERM UMR-S 1270, F75005, Paris, France; Sorbonne Université, F75005, Paris; Institut du Fer à Moulin, F75005, Paris, France

[4] European Homograft Bank, Brussels, Belgium

[5] Department of Thoracic Surgery, Nouvel Hôpital Civil, Hôpitaux de Strasbourg, Strasbourg, France

[6] AP-HP Centre, Université de Paris, Hôpital Cochin, Service Thoracique, Paris, France

[7] Centre de Recherche Clinique, Grand Hôpital de l'Est Francilien, Meaux, France

[8] INSERM-S 942, Centre for Biological Resources BB-0033-00064, University Paris, Hôpital Lariboisière, APHP Paris, France

SUPPLEMENTAL FIGURES

Supplemental Figure S1. Comparative analysis of 5-HT_{2B}R expressions between the different sites (aortic, mitral and tricuspid) for all 28 study patients (patient numbers 3126 to 3180). Using multivariate regression analysis for 5-HT_{2B}R levels, we found a correlation between the level of expression in the aortic and tricuspid positions (coefficient +0.75 [+0.17; +1.34]; *P*=0.0042) (n=14, Spearman's Rank Correlation), but no correlation for the mitral position (coefficient +0.077 [-0.38; +0.54]; *P*=0.71) (n=28, Spearman's Rank Correlation). We did not find any statistically significant correlation for any other 5-HTRs (Spearman's Rank Correlation) (data not shown), so that 5-HT_{2B}Rs were the only ones to be regulated between the different sites.

Supplemental Figure S2. The most significant statistical correlations for 5-HTR levels between pairs of 5-HTRs (one to another) based on multivariate regression analyses with explicative variables being other receptors (one to another) and implantation sites (i.e. aortic/mitral or tricuspid).

Supplemental Figure S2 is based on **Table 1.2.2**.

Figure S2 reports only those correlations between 5-HTR levels with a *P* value below or equal to 0.1 following multivariate regression analysis. Each coefficient between 5-HTRs is obtained after analysis of 70 samples. The width of the arrows is directly proportional to the level of statistical association based on multivariate analysis. Blue arrows correspond to a correlation between 5-HTRs where the coefficient has an absolute value between 0 and 1 and is positive. Red arrows correspond to a correlation where the coefficient has an absolute value between 0 and 1 and is negative.

There were strong correlations between 5-HT_4R and 5-HT_7R levels. There were also strong correlations revealed by multivariate analysis between 5-HT_{1A}Rs and 5-$HT_{1B/D}$Rs with a negative correlation between 5-HT_{1A}Rs and 5-$HT_{1B/D}$Rs. There were no significant correlations between the different 5-HT_2Rs (i.e. 5-HT_{2A}Rs, 5-HT2BRs or 5-HT_{2C}Rs). Finally, strong negative correlations were observed between 5-HT_1Rs levels (5-HT_{1A}Rs and 5-$HT_{1B/D}$Rs) and 5-HT_4Rs and 5-HT_7Rs levels.

Supplemental Figure S3. Hypothetical mechanisms linking non-5-HTR signaling and the signaling of the two main receptors associated with valvular pathology: 5-HT_2Rs (5-HT_{2A}R and 5-HT_{2B}R) based on Table 1.2.2 and Table 2.

In this study, we simply look at correlations between 5-HTRs and not at the direct effect of receptors on the levels of other 5-HTRs (inhibition or stimulation), with no evaluation of causality. Thus all these mechanisms are hypothetical. Also, we did not investigate any specific signaling, so that all the mechanisms developed later remain hypothetical.

We observed the presence of high levels of 5-HT_{2A}R, 5-HT_{2B}R and 5-HT_4R on human heart valves with almost the same quantities. With regard to 5-HTRs, in most cases in the different tissues analyzed, the expression of these receptors has been shown to be associated with increased signaling. Unlike other 5-HTRs and HT_{2A}R, the 5-HT_{2B}R family has been shown to play a key role in human valvular pathologies (2) (1, 3, 4) and G proteins (Gq/G11). Their subsequent signaling (PLC/DAG/IP3, Src/TG-β and Ras) has also been shown to be involved. As shown in Supplemental Figure S3, increased intracellular cAMP is theoretically associated with a decrease in Gq/G11 signaling through the stimulation of PKA that inhibits PLC, inhibiting ERK1/2 and the Smad pathways. In our study, we reported for the first time the presence of 5–HT_4R, which is known to be associated with a Gs protein and thus is supposed to increase cAMP and therefore inhibit 5-HT_2Rs signaling. By means of multivariate regression analysis (see Table 2), 5-HT_4R levels were statistically associated with the following: 5-HT7R (coef.=+1.43; [+1.28;+1.58] ; P<0.000001), 5-HT_{1A}R (coef.=+0.07; [+0.02; +0.12] P=0.0087) and a statistical tendency of association for 5-HT_{2B}R (coef=-0.06; P=0.076). Thus, there were positive correlations between 5-HT_7R and 5-HT_4R and negative correlations between 5-HT_{1A}R and 5-HT_{2B}R levels. Beside 5-HT_2Rs, 5-$HT_{1\,B/D}$R has been shown so far to be the only 5-HT_1Rs to be present, and functional,0 in human leaflets.

5-HTRs are known to be associated with 3 types of G proteins (i.e. Gi/G0, Gs or Gq/G11) depending on the type of 5-HTR (5). Hypothetical signaling is shown in yellow boxes. In mice, 5-HTR receptors (5-HT_{2A}R and 5-HT_{2B}R) have been shown to be expressed in neural crest cells during the valvular formation, but not 5-HT_{2C}Rs (6). The dose for blocking 5-HT_{2A}Rs was 10 times higher than that required for 5-HT_{2B}Rs. More recently, 5-HT_{2A}Rs and 5-HT_{2B}Rs have been shown to play a critical role in animal valvular pathologies (7) (4, 8) as well as in human pathologies (1, 3, 4). The enzyme involved in the peripheral synthesization of 5-HT (tryphophan hydroxylase 1 (TPH1)) has been shown to be present in normal valves and also to be increased in animal and human degenerative myxomatous valves (9) (7, 10). 5-HT_{2A}Rs, 5-HT_{2B}Rs and TPH1 can be induced by mechanical stimuli and the antagonists for each of these factors can block the valvular remodeling in vitro induced by mechanical stress, at least at the beginning (7).

Classical signaling of 5-HTR receptors involves activation of Gq/G_{11} and subsequent activation of PLC, and then DAG-PKC-ERK1/2-pERK or IP3- calcium (11). 5-HT_2Rs can also activate Src and Ras (orange Box) (12). Src is also associated with initial activation of the TGF-β receptor (13). Activation of the TGF-β receptor leads to activation of the canonical Smad 2/3/4 pathway. Activation of TGF-β canonical Smad 2/3/4 in heart valves is associated with the initial phases of fibrosis but also plays a physiological role in valve maintenance (i.e. green vertical bar) (14). Serotonin (i.e. 5-HT) plays a critical role in heart valve pathology (1, 3, 12, 15). The transporter for serotonin is expressed in heart valves during valve development (16) and later with a lower level of expression. In mice, a deficit of SERT is accompanied by valvular fibrosis, due to prolonged stimulation of the 5-HTRs (17). In animals, Serotonin SERT is down-regulated in the later stages of the disease, as has been observed in humans (1, 3, 4, 8).

The serotonin (5-HT) (i.e. **yellow circle**) (18) (upper part of **Supplementary Figure S3**) in the extracellular environment can interact with the 5-HTRs or interact with the SERT (i.e. serotonin transporter) and directly penetrates the cell without degradation. The enzyme involved in 5-HT synthesis, (i.e., the tryptophan hydroxylase 1 (TPH1)), has recently been shown to be present in human heart valve cusps (7, 10) and its activity is increased in heart valve pathology and/or by mechanical stimulation (1, 7). Inside the cell, the 5-HT can induce intra-cellular signaling by covalent fixation on glutamine residues through the transglutaminase-2 (TG2) enzyme. The TG2 multifunction enzyme is a member of the transglutaminase family with the calcium-dependent protein crosslinking in the same way as transamidation reactions provoke serotonylation of filamin A. Serotonylated filamin A will bind with actin cytoskeletal proteins that are involved in integrin-β inside/outside signaling. The last signal is necessary for proper ECM condensation and organization of heart valve architecture (18). Filamin A activates Smad 2/3, MAPK-P38 or ERK1/2 –pERK. 5-HT and can be degraded by the mitochondrial monoamine oxidase A (MAO-A) that is present in human valves, resulting in its main degradation product, (i. e., the 5-hydroxyindole acetic acid (5-HIAA)), which activates ROSs (12, 19). pERK signaling is associated with mitogenesis, DNA synthesis, cell cycle progression and proliferation. P38, MAPK and pERK are associated with ECM remodeling differentiation, migration and matrix condensation, ROS signaling with apoptosis, and oxidative damage (12). There is cross-talk between TPH1, 5-HIAA and Wnt. In animals, cyclic stretch has been shown to enhance 5-HT_{2A}Rs and 5-HT_{2B}Rs but not the transporter SERT. Valvular remodeling can be prevented by inhibitors of the 5-HT_2R family (20).

The Gi/G0 G proteins are associated with 5-HT_{1A}Rs and 5-$HT_{1B/D}$Rs and thus inhibit the AMP cyclase enzyme, resulting in a decrease in cAMP (i.e. purple bar). Moreover, 5-$H_{1B/D}$Rs have been shown to activate GIRK and ERK1/2 signaling in heart valves.

cAMP is known to directly inhibit ERK1/2 and Smad 2/3/4, and to activate Notch and PKA, a major mediator of cAMP activity (21). In normal human aortic valves, it has been shown that notch signaling is permanently activated and continuously inhibits Run X, which normally facilitates calcification (22, 23). PKA is known to inhibit PLC and will inhibit an important cascade of 5-HT_2R activation. PKA is also known to inhibit Rho signaling, which is especially present in stress and Rap1 (21). Integrins are cell membrane surface mechanoreceptors that are crucial for interactions with extracellular matrix proteins. PKA enhances Rap1, which is crucial for the integrin activation state (24). Mechanical stress alone has been shown to enhance not only the expression of 5-HT_{2A}Rs, and 5-HT_{2B}Rs but also TPH1 and SERT (7). Thus, there are close regulatory ties between 5-HTRs and mechanical stimuli. In valvular maturation and pathologies, it has been shown that TGF-β and Smad 5/6/7 signaling, instead of Smad 2/3/4 signaling, is likely to be predominant as well as the BMP and Wnt pathway activity (14).

FIGURES

Supplemental Figure S1

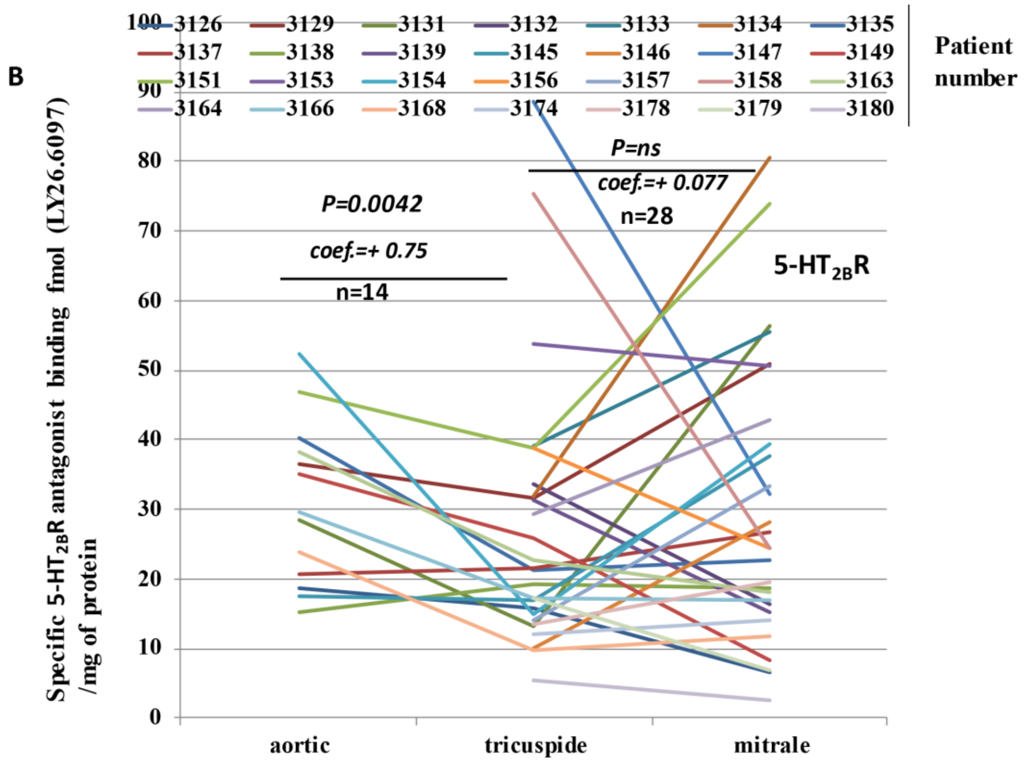

Levels of 5-HT$_{2B}$Rs for the differentspatients (i.e. numbers: 3126 to 3180) foraortic/ tricuspidand mitrale at the same time andcompared usingmultivariateregressionanalysis

Supplemental Figure S2 **Significative Correlations at mutivariate regressionanalyses for 5-HTRs levels one to another based on Table 1.2.2**

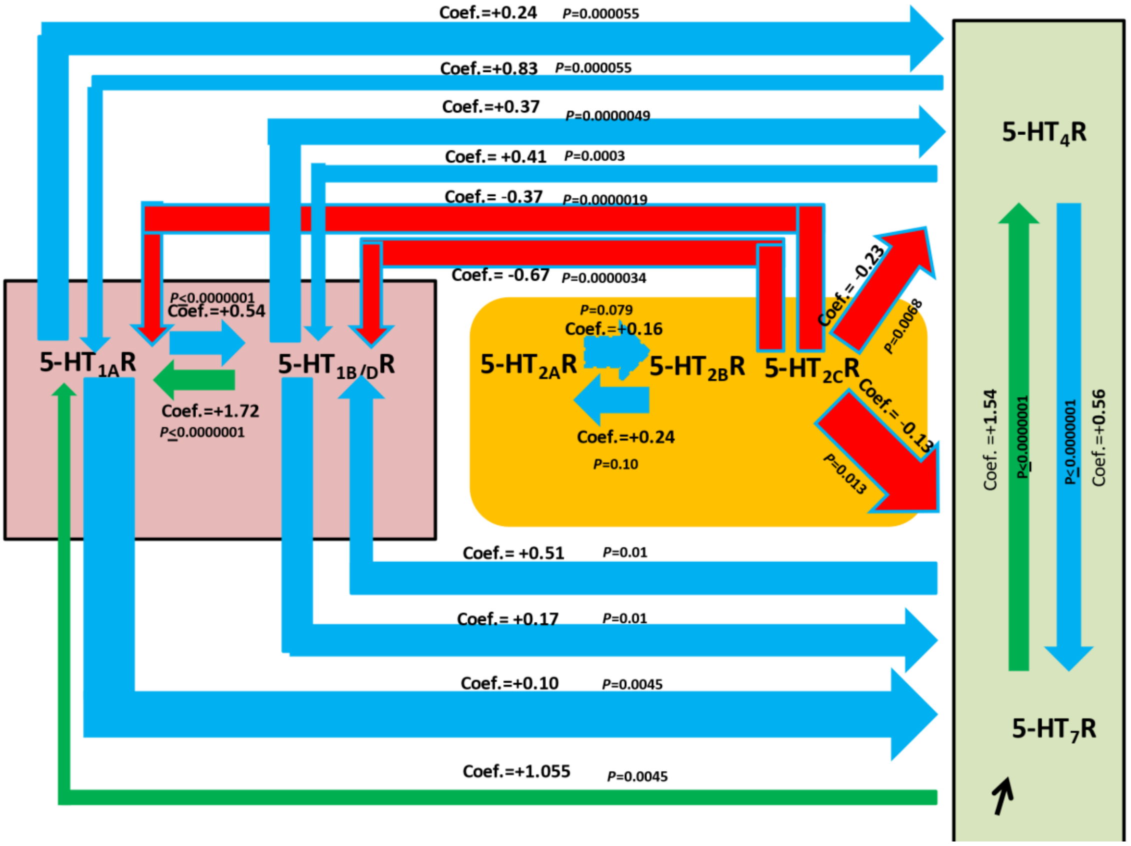

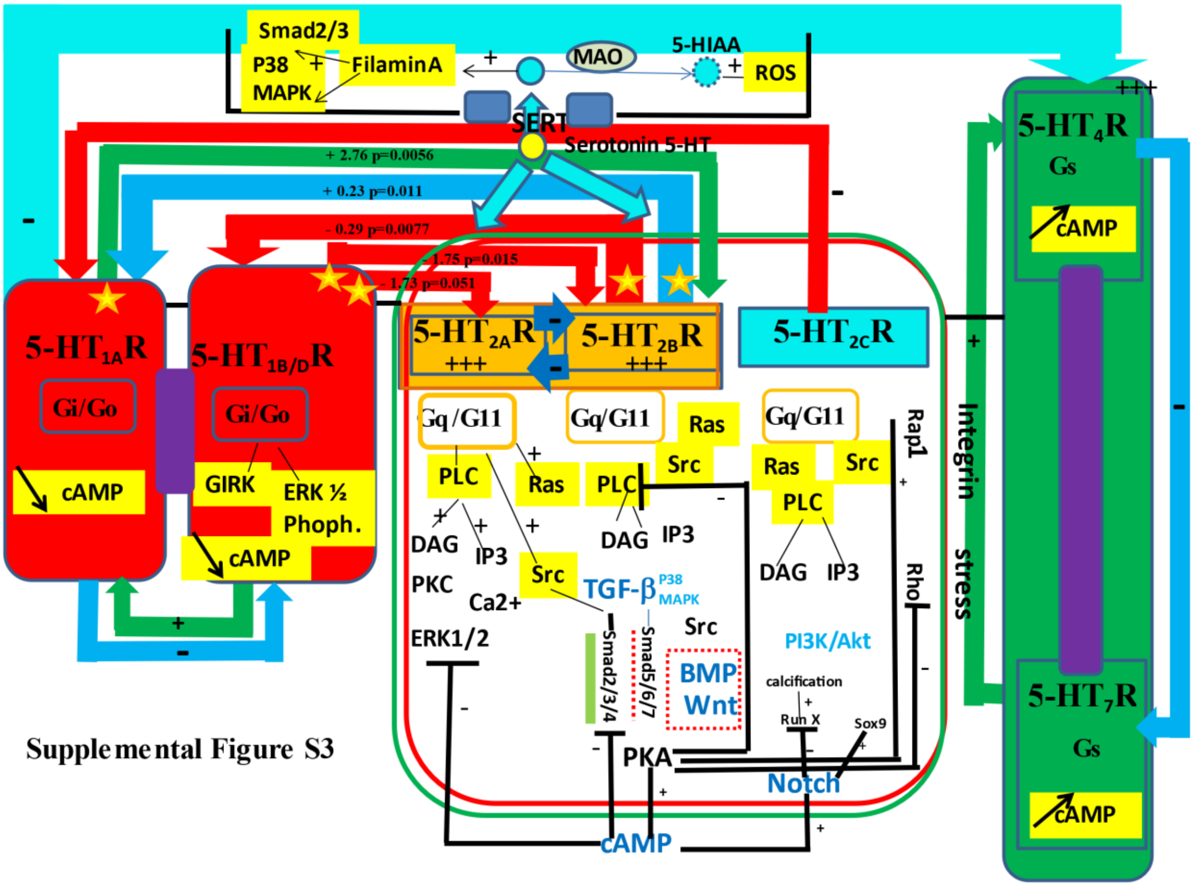

Supplemental Figure S3

Hypothetical interactions between non-5-H2TRs (5-H1AR, 5HT1B/DR, 5HT4R and 5-HT7R) and 5-HT2Rs (5-HT2AR and 5-HT2BR) in humans. 5-HT2AR and 5-HT2BR receptors and their pathways are the two mean 5-HTRs of animal and human valvular pathologies.

REFERENCES

1. K. H. Driesbaugh, E. Branchetti, J. B. Grau, S. J. Keeney, K. Glass, M. A. Oyama, N. Rioux, S. Ayoub, M. S. Sacks, J. Quackenbush, R. J. Levy and G. Ferrari: Serotonin receptor 2B signaling with interstitial cell activation and leaflet remodeling in degenerative mitral regurgitation. *J Mol Cell Cardiol*, 115, 94-103 (2018) doi:S0022-2828(17)30370-X [pii]
10.1016/j.yjmcc.2017.12.014
2. J. H. Fortier, B. Pizzarotti, R. E. Shaw, R. J. Levy, G. Ferrari and J. B. Grau: Durg-associated valvular heart diseases and serotonin-related pathways: a meta analysis. *Heart*, 105(5), 1140-1148 (2019) doi:10.2236/heartjnl-2018-314403
3. N. M. Thalji, M. A. Hagler, H. Zhang, G. Casaclang-Verzosa, A. A. Nair, R. M. Suri and J. D. Miller: Nonbiased Molecular Screening Identifies Novel Molecular Regulators of Fibrogenic and Proliferative Signaling in Myxomatous Mitral Valve Disease. *Circ Cardiovasc Genet*, 8(3), 516-28 (2015) doi:CIRCGENETICS.114.000921 [pii]
10.1161/CIRCGENETICS.114.000921
4. R. J. Levy, E. Fitzpatrick, E. Castillero, H. J. Shukla, V. V. Inamdar, A. E. Aghali, J. B. Grau, N. Rioux, S. J. Stachelek, C. Brown, A. M. Krieger and G. Ferrari: Inhibition and down regulation of the serotonin transporter contribute to the progression of degenerative mitral regurgitation. *bioRxiv* (2020)
5. J. D. McCorvy and B. L. Roth: Structure and function of serotonin G protein-coupled receptors. *Pharmacol Ther*, 150, 129-42 (2015) doi:S0163-7258(15)00029-7 [pii]
10.1016/j.pharmthera.2015.01.009
6. I. Moutkine, E. Quentin, B. P. Guiard, L. Maroteaux and S. Doly: Heterodimers of serotonin receptor subtypes 2 are driven by 5-HT2C protomers. *J Biol Chem*, 292(15), 6352-6368 (2017) doi:M117.779041 [pii]
10.1074/jbc.M117.779041
7. C. M. Lacerda, H. B. Maclea, J. D. Kisiday and E. C. Orton: Static and cyclic tensile strain induce myxomatous effector proteins and serotonin in canine mitral valves. *J Vet Cardiol*, 14(1), 223-30 (2012) doi:S1760-2734(12)00007-0 [pii]
10.1016/j.jvc.2011.12.002
8. C. C. Lu, M. M. Liu, G. Culshaw, M. Clinton, D. J. Argyle and B. M. Corcoran: Gene network and canonical pathway analysis in canine myxomatous mitral valve disease: a microarray study. *Vet J*, 204(1), 23-31 (2015) doi:S1090-0233(15)00095-7 [pii]
10.1016/j.tvjl.2015.02.021
9. S. Disatian, C. Lacerda and E. C. Orton: Tryptophan hydroxylase 1 expression is increased in phenotype-altered canine and human degenerative myxomatous mitral valves. *J Heart Valve Dis*, 19(1), 71-8 (2010)
10. A. Hulin, C. Deroanne, C. Lambert, J. O. Defraigne, B. Nusgens, M. Radermecker and A. Colige: Emerging pathogenic mechanisms in human myxomatous mitral valve: lessons from past and novel data. *Cardiovasc Pathol*, 22(4), 245-50 (2013) doi:S1054-8807(12)00147-0 [pii]
10.1016/j.carpath.2012.11.001
11. J. Xu, B. Jian, R. Chu, Z. Lu, Q. Li, J. Dunlop, S. Rosenzweig-Lipson, P. McGonigle, R. J. Levy and B. Liang: Serotonin mechanisms in heart valve disease II: the 5-HT2 receptor and its signaling pathway in aortic valve interstitial cells. *Am J Pathol*, 161(6), 2209-18 (2002) doi:S0002-9440(10)64497-5 [pii]
10.1016/S0002-9440(10)64497-5
12. E. Ayme-Dietrich, R. Lawson, S. Da-Silva, J. P. Mazzucotelli and L. Monassier: Serotonin contribution to cardiac valve degeneration: new insights for novel therapies? *Pharmacol Res*, 140, 33-42 (2019) doi:S1043-6618(18)30587-5 [pii]
10.1016/j.phrs.2018.09.009
13. J. J. Worthington, J. E. Klementowicz and M. A. Travis: TGFbeta: a sleeping giant awoken by integrins. *Trends Biochem Sci*, 36(1), 47-54 (2011) doi:S0968-0004(10)00165-9 [pii]
10.1016/j.tibs.2010.08.002

14. S. Chopra, N. Al-Sammarraie, Y. Lai and M. Azhar: Increased canonical WNT/beta-catenin signalling and myxomatous valve disease. *Cardiovasc Res*, 113(1), 6-9 (2017) doi:cvw236 [pii]
10.1093/cvr/cvw236
15. E. Ayme-Dietrich, R. Lawson, F. Cote, C. de Tapia, S. Da Silva, C. Ebel, B. Hechler, C. Gachet, J. Guyonnet, H. Rouillard, J. Stoltz, E. Quentin, S. Banas, F. Daubeuf, N. Frossard, B. Gasser, J. P. Mazzucotelli, O. Hermine, L. Maroteaux and L. Monassier: The role of 5-HT2B receptors in mitral valvulopathy: bone marrow mobilization of endothelial progenitors. *Br J Pharmacol*, 174(22), 4123-4139 (2017) doi:10.1111/bph.13981
16. L. M. Pavone, A. Spina, R. L. Muto, D. Santoro, V. Mastellone and L. Avallone: Heart valve cardiomyocytes of mouse embryos express the serotonin transporter SERT. *Biochem Biophys Res Commun*, 377(2), 419-422 (2008) doi:S0006-291X(08)01937-2 [pii]
10.1016/j.bbrc.2008.09.152
17. A. Mekontso-Dessap, F. Brouri, O. Pascal, P. Lechat, N. Hanoun, L. Lanfumey, I. Seif, N. Benhaiem-Sigaux, M. Kirsch, M. Hamon, S. Adnot and S. Eddahibi: Deficiency of the 5-hydroxytryptamine transporter gene leads to cardiac fibrosis and valvulopathy in mice. *Circulation*, 113(1), 81-9 (2006) doi:CIRCULATIONAHA.105.554667 [pii]
10.1161/CIRCULATIONAHA.105.554667
18. L. M. Pavone and R. A. Norris: Distinct signaling pathways activated by "extracellular" and "intracellular" serotonin in heart valve development and disease. *Cell Biochem Biophys*, 67(3), 819-28 (2013) doi:10.1007/s12013-013-9606-8
19. R. A. Pena-Silva, J. D. Miller, Y. Chu and D. D. Heistad: Serotonin produces monoamine oxidase-dependent oxidative stress in human heart valves. *Am J Physiol Heart Circ Physiol*, 297(4), H1354-60 (2009) doi:00570.2009 [pii]
10.1152/ajpheart.00570.2009
20. K. Balachandran, M. A. Bakay, J. M. Connolly, X. Zhang, A. P. Yoganathan and R. J. Levy: Aortic valve cyclic stretch causes increased remodeling activity and enhanced serotonin receptor responsiveness. *Ann Thorac Surg*, 92(1), 147-53 (2011) doi:S0003-4975(11)00742-9 [pii]
10.1016/j.athoracsur.2011.03.084
21. P. Sassone-Corsi: The cyclic AMP pathway. *Cold Spring Harb Perspect Biol*, 4(12) (2012) doi:4/12/a011148 [pii]
10.1101/cshperspect.a011148
22. J. Chen, L. M. Ryzhova, M. K. Sewell-Loftin, C. B. Brown, S. S. Huppert, H. S. Baldwin and W. D. Merryman: Notch1 Mutation Leads to Valvular Calcification Through Enhanced Myofibroblast Mechanotransduction. *Arterioscler Thromb Vasc Biol*, 35(7), 1597-605 doi:ATVBAHA.114.305095 [pii]
10.1161/ATVBAHA.114.305095
23. V. Garg, A. N. Muth, J. F. Ransom, M. K. Schluterman, R. Barnes, I. N. King, P. D. Grossfeld and D. Srivastava: Mutations in NOTCH1 cause aortic valve disease. *Nature*, 437(7056), 270-4 (2005) doi:nature03940 [pii]
10.1038/nature03940
24. T. Bromberger, L. Zhu, S. Klapproth, J. Qin and M. Moser: Rap1 and membrane lipids cooperatively recruit talin to trigger integrin activation. *J Cell Sci*, 132(21) (2019) doi:jcs.235531 [pii]
10.1242/jcs.235531
